# Prospektive Untersuchung der extrakraniellen Duplexsonographie zum Nachweis des zerebralen Perfusionsstillstands bei Patienten mit irreversiblem Hirnfunktionsausfall

**DOI:** 10.1007/s00115-023-01521-4

**Published:** 2023-07-21

**Authors:** Johann Lambeck, Christoph Strecker, Wolf-Dirk Niesen, Jürgen Bardutzky

**Affiliations:** 1 Klinik für Neurologie und Neurophysiologie, Universitätsklinikum Freiburg, Freiburg, Deutschland; 2 Klinik für Neurologie und Neurophysiologie, Universitätsklinikum Freiburg, Breisacherstr. 64, 79106 Freiburg, Deutschland

**Keywords:** Hirntod, Zusatzverfahren, Ultraschall, EEG, Bettseitig, Brain death, Ancillary test, Ultrasound, EEG, Bedside

## Abstract

**Hintergrund:**

Eine breitere Verfügbarkeit der bettseitig einsetzbaren farbkodierten Duplexsonographie („colour-coded duplex sonography“ [CCD]) zum Nachweis des zerebralen Perfusionsstillstands (ZP) wäre wichtig, um den Einsatz bei der Diagnostik des irreversiblen Hirnfunktionsausfalls (IHA-Dx) zu verbessern.

**Fragestellung:**

Ist die extrakranielle CCD der üblichen transkraniellen CCD der hirnversorgenden Gefäße (ECCD vs. TCCD) zum Nachweis des ZP bei der IHA-Dx bez. Spezifität und Sensitivität gleichwertig?

**Material und Methoden:**

Studienzeitraum 01/19 bis 06/22. Screening von 136, Einschluss von 114 Patienten mit schweren Hirnläsionen > 24 h nach dem Auftreten weiter, lichtstarrer Pupillen, Apnoe und abgeschlossener IHA-Dx. Ausschluss von Patienten ohne Hirnstammareflexie und ohne richtlinienkonforme Einsetzbarkeit der CCD. Ergänzende ECCD (und ggf. TCCD bei Einsatz einer anderen Methode zum Irreversibilitätsnachweis).

**Ergebnisse:**

Feststellung IHA (IHA+) in 86,8 % (99/114), kein IHA (IHA−) in 13,2 % (15/114). Die ECCD war in allen Fällen vollständig durchführbar, in 94/99 IHA+-Fällen fand sich ein zum ZP passender Befund (ECCD+), in 5 Fällen nicht (ECCD−). Alle 15 Patienten mit IHA− zeigten einen ECCD−-Befund. Somit lag die Spezifität der ECCD bei 1,0, die Sensitivität bei 0,949. Die TCCD zeigte in 56 Fällen einen ZP (TCCD+), bei allen fand sich auch ECCD+. Einen inkonklusiven Befund der TCCD bei IHA+ zeigten 38 Fälle, parallel ECCD+ bei all diesen. In 20 Fällen war der TCCD ohne Nachweis des ZP (TCCD−) und auch ECCD−. 15 dieser Patienten waren IHA−, 5 IHA+.

**Diskussion:**

Die TCCD war in einem Drittel der Fälle nicht komplett durchführbar oder inkonklusiv, die ECCD war dagegen immer durchführbar. Die ECCD zeigte eine hohe Validität bez. des Nachweises des ZP. Daher sollte ein alleiniger Einsatz der ECCD zum Nachweis des ZP bei der IHA-Dx diskutiert werden.

## Abstract (en)

**Background:**

A broader distribution of bedside color-coded duplex sonography (CCD) for detection of cerebral circulatory arrest (CCA) would be important to improve its use in the diagnosis of irreversible loss of brain function (ILBF-Dx).

**Question:**

Is extracranial compared to the commonly applied transcranial CCD of the brain-supplying vessels (ECCD vs. TCCD) equivalent for the detection of CCA in ILBF-Dx regarding specificity and sensitivity?

**Material and methods:**

Study period January 2019–June 2022, screening of 136 and inclusion of 114 patients with severe brain lesions > 24 h after onset of fixed and dilated pupils, apnea and completed ILBF-Dx. Exclusion of patients without brainstem areflexia and guideline-conform applicability of CCD. Complementary ECCD (and TCCD, if other method used for irreversibility detection).

**Results:**

Detection of ILBF (ILBF+) in 86.8% (99/114), no ILBF (ILBF−) in 13.2% (15/114). ECCD was fully feasible in all patients; findings matching CCA were found in 94/99 ILBF+ cases (ECCD+) and not in 5 patients (ECCD−). All 15 patients with ILBF− showed ECCD− findings. Thus, the specificity of ECCD was 1.0, and the sensitivity was 0.949. TCCD showed CCA in 56 patients (TCCD+), and ECCD+ was also found in all of them. An inconclusive result of TCCD in ILBF+ was found in 38 cases, with parallel ECCD+ in all of these patients. In 20 cases, TCCD did not show CCA (TCCD−), these also showed ECCD−. Of these patients 15 were ILBF− and 5 were ILBF+.

**Discussion:**

TCCD was not completely feasible or inconclusive in one third of the cases, whereas ECCD was always feasible. ECCD showed high validity with respect to the detection of CCA. Therefore, the possibility of using ECCD alone to detect CCA in ILBF-Dx should be discussed.

Zusatzverfahren, die im Rahmen der Diagnostik des irreversiblen Hirnfunktionsausfalls (IHA) eingesetzt werden, sollten (i) einfach durchzuführen und (ii) eindeutig und zuverlässig zu interpretieren sein sowie (iii) den Patienten nicht gefährden. Nach deutschen und internationalen Richtlinien ist der Nachweis des zerebralen Perfusionsstillstandes eine Möglichkeit, die Irreversibilität des Hirnfunktionsausfalls nachzuweisen. Unter Berücksichtigung des eingangs Erwähnten stellt hierbei die Sonographie das einzige bettseitig durchführbare Verfahren dar. Bei diesem Patientenkollektiv kann allerdings die transkranielle Sonographie aus verschiedenen Gründen erschwert sein. In dieser Studie wurde untersucht, ob eine > 24 h nach Auftreten der ersten Symptome des IHA durchgeführte technisch einfachere alleinige extrakranielle Duplexsonographie eine Alternative sein könnte, die o. g. Anforderungen erfüllt.

## Hintergrund

Bei der Diagnostik des irreversiblen Hirnfunktionsausfalls („Hirntoddiagnostik“) werden in vielen Ländern Zusatzverfahren zur Beurteilung der Irreversibilität des klinischen Syndroms eingesetzt oder sind sogar vorgeschrieben [6, 9, 12].

Ziel dieser Zusatzverfahren ist es, entweder den Ausfall der Hirnfunktion (EEG, evozierte Potenziale) oder einen Stillstand der Hirnperfusion (Doppler‑/Duplexsonographie der Hirnbasisarterien, CT-Angiographie, digitale Subtraktionsangiographie, SPECT) nachzuweisen.

Die diesbezügliche deutsche Richtlinie [1, 14, 19] fordert den Nachweis des zerebralen Perfusionsstillstands (ZP) in Fällen, bei denen die klinische Untersuchung und hier vor allem der Apnoetest nicht vollständig durchführbar oder potenziell kompromittiert ist. Beispiele hierfür sind hypotherme Patienten, Patienten mit relevanter COPD oder relevanten Spiegeln von Analgosedativa. Zudem kann durch den Nachweis eines ZP (ab dem vollendeten 3. Lebensjahr) auf die 2. klinische Untersuchung nach 12–72 h bei rein supratentorieller primärer und bei sekundärer Hirnschädigung verzichtet werden.

Das einzige hierfür in der Routine bettseitig einsetzbare Verfahren ist die transkranielle Doppler- und Duplexsonographie (TCD bzw. TCCD). Nach der Richtlinie der Bundesärztekammer (BÄK; [14]) müssen beim Einsatz der TCCD „intrakraniell die M1-Segmente der Aa. cerebri mediae, die Aa. carotides internae, die V4-Segmente der Aa. vertebrales und die A. basilaris sowie eventuell detektierbare weitere Hirnbasisarterien untersucht werden“. In diesen Gefäßabschnitten müssen die Zeichen des ZP (biphasische Strömungssignale oder frühsystolische Spitzen) nachgewiesen werden. Wenn intrakraniell keine Strömungssignale ableitbar sind, kann dies nur als Zeichen eines ZP gewertet werden, „wenn derselbe Untersucher bei gleicher Geräteeinstellung bei einer früheren Untersuchung eindeutig ableitbare intrakranielle Strömungssignale dokumentiert hat oder wenn an den extrakraniellen hirnversorgenden Arterien die Zeichen des zerebralen Kreislaufstillstandes nachweisbar sind.“ Im klinischen Alltag erfolgt bei nicht nachweisbaren Strömungssignalen einzelner oder aller intrakranieller Gefäße in der TCCD in diesem Kontext allerdings häufig ein Methodenwechsel, eine primär ausschließliche Untersuchung der extrakraniellen hirnversorgenden Gefäße wird durch einige Autoren nicht befürwortet und ist bisher auch noch nie separat untersucht worden [3]. In einer vorausgehenden Studie unserer Arbeitsgruppe zu diesem Thema wurde die Validität der ECCD gegen das EEG (Goldstandard) getestet und hohe Werte für Sensitivität und Spezifität bez. des Nachweises der Irreversibilität eines Hirnfunktionsausfalls gefunden [10].

Die praktische Durchführung der TCCD ist weiterhin durch folgende Aspekte erschwert:

i. Notwendigkeit einer speziellen Ausbildung,
ii. Vorhandensein einer transkraniellen Ultraschallsonde bzw. des entsprechenden Settings,
iii. Vorhandensein eines transtemporalen Schallfensters,
iv. erschwerte Auffindbarkeit der intrakraniellen Gefäße im Unterschied zur extrakraniellen Duplexsonographie aufgrund der fehlenden direkten Visualisierung bei gleichzeitig bei diesem Patientenkollektiv häufig vorliegender Schwellung des Hirngewebes mit veränderter Neuroanatomie und Lage der Gefäße,
v. fehlender Signalnachweis aufgrund der Ausprägung der zugrunde liegenden intrakraniellen Drucksteigerung (siehe vergleichende Studien zur Angiographie).

Dies unterstreicht die Wichtigkeit, den Ultraschall der hirnversorgenden Gefäße in diesem Kontext zu vereinfachen.

Pathophysiologisch ist naheliegend, dass der Nachweis der Zeichen des Perfusionsstillstands in den extrakraniellen Abschnitten der Aa. carotides internae und vertebrales dies indirekt auch für die sich distal anschließenden intrakraniellen Gefäßabschnitte (i.e. ZP) anzeigt. Der alleinige Nachweis des Perfusionsstillstands extrakraniell mittels ECCD könnte dann zum Nachweis der Irreversibilität des Hirnfunktionsausfalls ausreichend sein.

In der vorliegenden prospektiven Studie wurde deshalb im Zeitraum 01/2019 bis 06/2022 bei allen Patienten mit (i) klinisch nachgewiesener Hirnstammareflexie und (ii) abgeschlossener IHA-Diagnostik nach Richtlinie der BÄK, bei denen (iii) der TCCD zum Irreversibilitätsnachweis richtliniengemäß zulässig war, immer sowohl eine TCCD als auch eine ECCD durchgeführt.

Falls der Nachweis eines ZP mittels TCCD nicht möglich, inkonklusiv oder negativ war, wurde ergänzend ein EEG für den Irreversibilitätsnachweis durchgeführt.

Im Rahmen dieser Studie sollte so geklärt werden, ob durch die alleinige Untersuchung der 4 extrakraniellen hirnversorgenden Gefäße der Nachweis eines ZP in einer höheren Anzahl der Fälle gelingt als bei der Untersuchung der nach Richtlinie vorgeschriebenen intrakraniellen Gefäßabschnitte mittels TCCD.

## Material und Methoden

### Studienpopulation

Eine Gruppe von 136 konsekutiven Patienten mit schweren Hirnschäden unterschiedlicher Ätiologie (Tab. 1) wurde zwischen Januar 2019 und Juni 2022 prospektiv auf das Vorliegen eines irreversiblen Hirnfunktionsausfalls untersucht. Einschlusskriterium für die Studie war eine nach Richtlinie der BÄK (4. Fortschreibung [14]) abgeschlossene IHA-Diagnostik. Ausschlusskriterien waren Faktoren, die die CCD nach Richtlinie potenziell beeinflussen (große Knochendefekte oder fehlendes pulsatiles Strömungsprofil bei Patienten an der venoarteriellen extrakorporalen Membranoxygenierung [vaECMO]). Außerdem wurden Patienten mit unvollständiger Hirnstammareflexie ausgeschlossen.

**Table 1:** 

Charakteristika	*n*
Alter [Jahre]	Median 56, IQR 45–67
Geschlecht [weiblich/männlich]	57/79
Gescreente Patienten	136
*Ätiologie*	
ICB	21
SAB	10
Ischämischer Schlaganfall	16
SHT	4
Hypoxie	40
Kombination	37
Andere	8
*Eingeschlossene Patienten*	*114*
Davon an der vaECMO	14
*Ausgeschlossene Patienten*	*22*
Inkomplette Hirnstammareflexie	9
Große Knochendefekte	8
Kardialer Eigenauswurf zu gering (vaECMO)	5
IHA bestätigt	99
IHA nicht bestätigt	15

*IQR* Interquartilsabstand, *ICB* intrazerebrale Blutung, *SAB* subarachnoidale Blutung, *SHT* Schädel-Hirn-Trauma, *Kombination* Kombinationen verschiedener Ätiologie, z. B. SAB und Hypoxie, *vaECMO* venoarterielle extrakorporale Membranoxygenierung, *IHA* irreversibler Hirnfunktionsausfall

### Klinische Untersuchung

Im Universitätsklinikum Freiburg wird der IHA gemäß den von der BÄK veröffentlichten Richtlinien von einem Team erfahrener Neurointensivmediziner diagnostiziert (d. h. Durchführung der Untersuchungen durch 2 Fachärzte für Neurologie, Zusatzbezeichnung neurologische Intensivmedizin, mehrere Jahre Erfahrung sowohl in der klinischen Untersuchung hirntoter Patienten als auch in der Durchführung der Zusatzverfahren in jeweils mehr als 100 Fällen). Dieses Team wird bei allen Intensivpatienten des Klinikums mit klinischem Verdacht auf IHA (d. h. schwere Hirnschädigung im Schädel-CT oder -MRT und vermuteter Hirnstammareflexie) konsultiert, meist im Zusammenhang mit der Frage einer möglichen Organspende, aber auch im Rahmen der „end of life care“ und damit verbundenen Entscheidungsfindung über die Fortsetzung oder Beendigung intensivmedizinischer Maßnahmen. Alle im Folgenden genannten, durch das Studienteam durchgeführten Untersuchungen erfolgten > 24 h nach der ersten Feststellung der o. g. klinischen Anzeichen des IHA durch die Stationsärzte der jeweiligen Intensivstationen.

Bei jedem Patienten wurde richtliniengemäß (i) zunächst eine gründliche Analyse hinsichtlich der Voraussetzungen des IHA durchgeführt und nach Prüfung alternativer Faktoren, die den komatösen Bewusstseinszustand des Patienten ganz oder teilweise erklären könnten, (ii) eine klinische Untersuchung der Hirnstammreflexe einschließlich Apnoetest durchgeführt.

Patienten mit unvollständiger Hirnstammareflexie und Patienten, bei denen die TCCD zum Irreversibilitätsnachweis nach Richtlinie (4. Fortschreibung) nicht zugelassen war (große Defekte des Schädelknochens oder zu niedriger kardialer Eigenauswurf [vaECMO], d. h. duplexsonographisch kein pulsatiles Flusssignal ableitbar), wurden von der Studienteilnahme ausgeschlossen.

### Studienuntersuchung (farbkodierte Duplexsonographie extra- und ggf. transkraniell)

Nach Abschluss der offiziellen IHA-Diagnostik wurde durch den bez. des Ergebnisses der offiziellen apparativen IHA-Diagnostik verblindeten 2. Untersucher eine CCD der extra- und (falls nicht bereits zum Irreversibilitätsnachweis eingesetzt) intrakraniellen hirnversorgenden Gefäße durchgeführt (Philips CX50® [Philips, Amsterdam, Niederlande] oder Toshiba [Toshiba, Tokio, Japan] Aplio® 400, L12‑3 Breitband-Linear-Array-Schallkopf, Frequenzspektrum 3–12 MHz und S5‑1 Breitband-Reinwellen-Sektor-Array-Schallkopf, Frequenzspektrum 1–5 MHz).

#### TCCD

Die Richtlinie der BÄK verlangt den Nachweis von entweder frühen systolischen Spitzen oder Pendelfluss mit gleichen antero- und retrograden Anteilen des Doppler-Zeit-Frequenzspektrums innerhalb eines Herzzyklus für > 30 min als Irreversibilitätsnachweis (d. h. Aufzeichnung der Flussprofile in u. g. Gefäßabschnitten zu Beginn und unmittelbar nach einer Zeitspanne von 30 min). Bei Patienten mit biphasischen Flusssignalen wurde darauf geachtet, dass nur schmale, monophasische Flusssignale (orthograde Komponente) als Zeichen des Perfusionsstillstands akzeptiert wurden. Vorgeschrieben ist ein mittlerer arterieller Druck von > 60 mm Hg. Bei Patienten, die dies nicht erfüllten, wurde Noradrenalin i.v. verabreicht, bis dies erreicht war.

Die TCCD umfasste die beidseitige Untersuchung der A. cerebri media (M1-Segmente), der A. carotis interna (ACI, C1-Segment), der A. vertebralis (VA, V4/5-Segment) sowie die Untersuchung der A. basilaris (BA) und aller anderen sichtbaren intrakraniellen Arterien.

#### ECCD

Die gleichen Maßstäbe wurden auch an die ECCD-Untersuchung angelegt, auch bez. der Richtlinienkonformität: Bei Patienten mit großen Knochendefekten kann eine regional begrenzte zerebrale Durchblutung vorliegen (z. B. über extra-/intrakranielle Anastomosen). Bei diesen Patienten kann der zerebrale Perfusionsstillstand gemäß den zum Untersuchungszeitpunkt gültigen Richtlinien zur Feststellung des IHA (4. Fortschreibung) nicht durch eine TCCD der basalen Hirnarterien nachgewiesen werden. Da für unsere Studienkohorte die gleichen Kriterien galten, wurden diese Patienten von der Teilnahme ausgeschlossen. Darüber hinaus war bei einigen Patienten unter vaECMO-Behandlung eine sonographische Beurteilung pulsatiler Flusssignale aufgrund des geringen Eigenauswurfs nicht möglich. Daher wurden 5 von insgesamt 14 Patienten unter vaECMO-Behandlung ebenfalls von der Studie ausgeschlossen.

Die ECCD umfasste die beidseitige Untersuchung der ACI (so weit distal wie möglich) und des V2/3-Segments der VA.

### Elektroenzephalographie (EEG)

Bei Patienten, bei denen die TCCD gar nicht oder nicht bei allen richtliniengemäß geforderten Gefäßabschnitten möglich bzw. inkonklusiv (TCCD?) war oder die Zeichen des ZP fehlten (TCCD−), wurde durch einen der Untersucher ergänzend ein EEG abgeleitet (Natus [Natus Neurology Inc., Middleton, WI, USA] Deltamed itmed®-Gerät mit Natus Neurofile®-Software auf einem Lenovo [Lenovo Ltd., Hongkong, China] ThinkPad®-Laptop, 23 Stahlnadelelektroden, 10–20 Platzierung, Elektrodenimpedanz 1–5 kΩ, Hochpassfilter 70 Hz, Tiefpassfilter 0,53–0. 16 Hz/Zeitkonstante 0,3–1 s, Verstärkung 2 μV/mm, wiederholte Applikation von Schmerzreizen im Gesicht und an den Extremitäten, auditive und visuelle Reize, zusätzliche Montage mit doppelter Elektrodendistanz, Ableitedauer > 30 min). Bei Artefakten im Sinne von Skalp-EMG-Restaktivität erfolgte die Gabe kurz wirksamer Muskelrelaxanzien (z. B. Rocuronium i.v.).

### Endpunkte

Primärer Endpunkt der Studie war der Nachweis der geforderten Flusssignale des ZP in allen 4 extrakraniellen hirnversorgenden Gefäßen bei Durchführung der Untersuchung > 24 h nach dem Auftreten der ersten klinischen Symptome eines IHA.

Weiterhin erfolgte die Überprüfung der Hypothese, dass der Nachweis der Flusssignale eines ZP bei reiner Untersuchung der extrakraniellen hirnversorgenden Gefäße in einer höheren Anzahl der Fälle gelingt als bei Untersuchung der nach Richtlinie vorgeschriebenen intrakraniellen Gefäßabschnitte mittels TCCD (H1, Alternativhypothese).

### Statistische Analyse und Datenpräsentation

Die Daten wurden nach Kolmogorov-Smirnov-Testung bei fehlender Normalverteilung als Median und Interquartilsabstand (IQR) angegeben. Der Nachweis des ZP in den 4 extrakraniellen hirnversorgenden Gefäßen wurde mit der Feststellung des IHA in der IHA-Diagnostik sowie den erfolgten apparativen Verfahren im Rahmen der IHA-Diagnostik mittels χ^2^-Test verglichen und die Spezifität, Sensitivität sowie der positive (PPV) und negative (NPV) prädiktive Wert berechnet. Alle statistischen Analysen wurden mit dem Softwarepaket IBM® SPSS® Statistics 21 (IBM Corporation, Armonk, NY, USA) durchgeführt.

## Ergebnisse

### Patientencharakteristika

**Figure 1:**
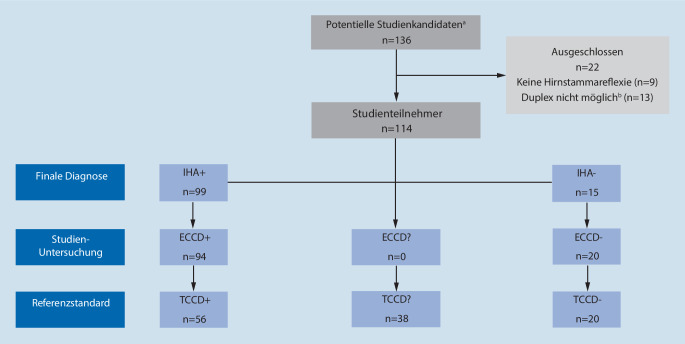

Von 01/2019 bis 06/2022 wurden insgesamt 136 IHA-Untersuchungen durchgeführt. Die Patientencharakteristika aller Patienten und weitere Details zu den Untersuchungen sind in Tab. 1 dargestellt, das Einschlussschema ist in Abb. 1 dargestellt.

Neun Patienten wurden aufgrund einer inkompletten Hirnstammareflexie ausgeschlossen, bei weiteren 13 Patienten war eine Sonographie nicht erlaubt (keine Richtlinienkonformität gemäß 4. Fortschreibung) oder nicht möglich. Dies war auf große Knochendefekte (*n* = 8) oder ein niedriges Herzzeitvolumen bei Patienten mit vaECMO (*n* = 5) zurückzuführen.

Somit wurden 114 Patienten mit nachgewiesener Hirnstammareflexie und richtliniekonformer Sonographie eingeschlossen.

### Primärer Endpunkt – Validität der ECCD

Die Validität der ECCD wurde im Vergleich mit dem Ergebnis der zum IHA passenden (IHA+) oder nichtpassenden (IHA−) 3‑stufigen IHA-Diagnostik bestimmt (Tab. 2). Diese war bei allen 114 Patienten mittels apparativen Irreversibilitätsnachweises erfolgt, entweder mit TCCD oder mit EEG.

**Table 2:** 

	IHA+	IHA−	Anzahl
ECCD+	94	–	94
ECCD−	5	15	20
Anzahl	99	15	114

*IHA+* finale Diagnose irreversibler Hirnfunktionsausfall liegt vor, *IHA-* finale Diagnose irreversibler Hirnfunktionsausfall liegt nicht vor, *ECCD+* Flussprofil passend zu Perfusionsstillstand, *ECCD–* Flussprofil nicht passend zu Perfusionsstillstand

**Figure 2:**
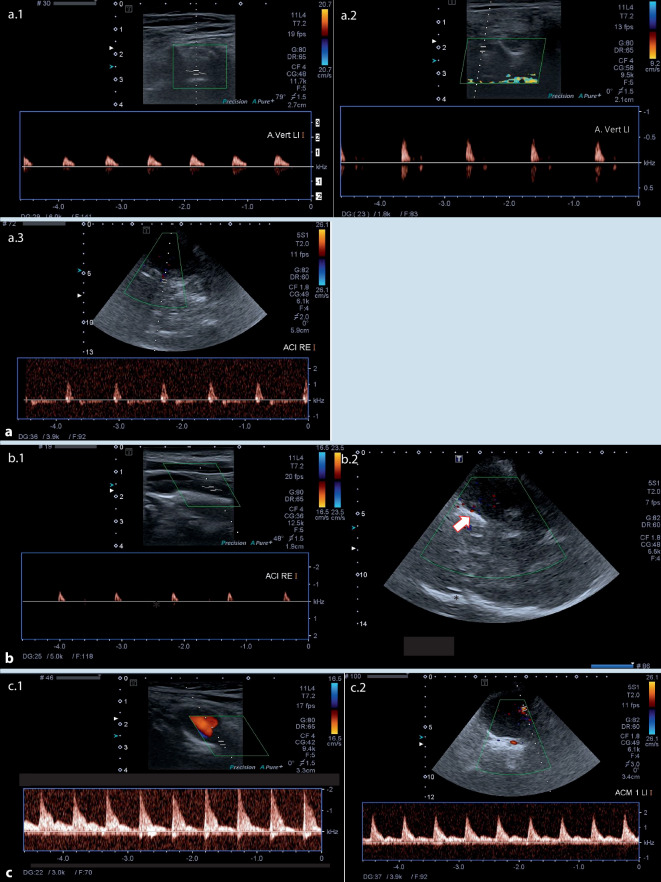

Von den 99 Patienten mit IHA+ zeigten 94 Patienten auch einen ECCD+ Befund und 5 Patienten einen ECCD− Befund (entsprechend 5 falsch-negativen Testergebnissen). Unter den 15 IHA− Patienten fand sich bei keinem der Patienten ein ECCD+ Befund und in allen 15 Patienten ein ECCD− Befund. Die ECCD zeigt eine Spezifität und einen PPV von 1,0, die Sensitivität liegt bei 0,949 und der NPV bei 0,75 (siehe auch Abb. 2).

### Prüfung der Alternativhypothese

Die Ergebnisse der TCCD und ECCD für alle 114 Patienten sind in Tab. 3 zusammengefasst.

**Table 3:** 

ECCD	TCCD	*N* = 114 *n* (%)
+	+	56 (49)
+	–	0
+	?	38 (33)
–	+	0
–	–	20 (18)
–	?	0
?	+/−/?	0

*TCCD+* bzw. *ECCD+* Flussprofil passend zu zerebralem Perfusionsstillstand nach Richtlinie, *TCCD-* bzw. *ECCD−* Flussprofil nichtpassend zu zerebralem Perfusionsstillstand, *TCCD?* bzw. *ECCD?* kein Schallfenster oder kein Gefäß/nicht alle richtliniengemäß geforderten Gefäße detektierbar oder inkonklusiv

Beispiele für alle drei gefundenen Konstellationen: ECCD+/TCCD+; ECCD+/TCCD?; ECCD−/TCCD− zeigt Abb. 2.

Bei den 99 Patienten mit IHA+ gelang der Nachweis eines ZP mittels ECCD signifikant häufiger als mittels TCCD (in 94 vs. 56 Fällen, *p* = 0,013), sodass die Alternativhypothese zutreffend ist.

- *TCCD+ und TCCD−:* Bei Nachweis eines ZP in der TCCD (i.e. TCCD+; 56 Patienten) zeigte sich in keiner ECCD eine Restperfusion. Ebenso konnte bei keinem Patienten mit Restperfusion in der TCCD (i.e. TCCD−; 20 Patienten) ein Perfusionsstillstand in der ECCD nachgewiesen werden. Es gab keinen Fall, bei dem die TCCD einen ZP, die ECCD aber eine Restperfusion zeigte (ECCD+/TCCD−) oder die TCCD eine Restperfusion, die ECCD aber einen Perfusionsstillstand (ECCD−/TCD+).
- *TCCD?:* Bei 38 der 114 Patienten (33 %) war die TCCD nicht oder nicht vollständig möglich bzw. nicht schlüssig (i.e. TCCD?). Dies war im Einzelnen darauf zurückzuführen, dass bei 10 Patienten kein ausreichendes Schallfenster vorhanden war, bei einem Patienten eine Schusswunde vorlag und bei 27 Patienten keine (*n* = 15) oder nicht alle (*n* = 12) geforderten intrakraniellen Gefäße gefunden werden konnten und bei 7 Patienten zusätzlich einzelne Gefäße mit erhaltenem diastolischem Fluss gefunden wurden. Bei diesen 38 Patienten erfolgte die Ableitung eines EEG, welches in allen Fällen keine zerebrale Aktivität anzeigte (d. h. Nulllinien-EEG). Bei den übrigen 76 Patienten lieferte die TCCD die gleichen Ergebnisse wie die ECCD (56 TCCD+, 20 TCCD−).
- *ECCD:* Die ECCD war bei allen 114 Patienten durchführbar. Bei 94 Patienten zeigte sich ein Perfusionsstillstand, bei keinem Patienten war in einem oder mehreren Gefäßen kein Flusssignal ableitbar.

Bei 20 Patienten ergab die ECCD keinen zum IHA passenden Befund (i.e. ECCD−), bei all diesen Patienten zeigte die TCCD ebenfalls keinen Perfusionsstillstand.

Bei diesen 20 Patienten erfolgte ergänzend ein EEG, welches in 15 Fällen mit der ECCD übereinstimmte und noch Restaktivität zeigte. In den verbleibenden 5 Fällen zeigte das EEG eine Nulllinie.

Bei allen 5 Patienten lag als Ursache der Hirnschädigung eine Hypoxie vor (5-mal post kardiopulmonaler Reanimation (CPR), 3 davon unter vaECMO-Therapie). Der Zeitpunkt, zu dem die IHA-Diagnostik bei diesen Patienten erfolgte, lag in allen Fällen > 72 h nach dem Auftreten der ersten klinischen Symptome des IHA (Median 74 h, IQR 4).

## Diskussion

In dieser Studie wurde bei Patienten mit IHA prospektiv untersucht, in wie vielen Fällen die Flusssignale eines zerebralen Perfusionsstillstands mittels extrakranieller Duplexsonographie bei Durchführung der Untersuchung > 24 h nach dem Auftreten der ersten klinischen Symptome des IHA und nach abgeschlossener IHA-Diagnostik nachweisbar sind.

### Durchführbarkeit

Die Anwendung der ECCD war bei allen 114 Patienten möglich und einfach durchführbar (auch wenn z. T. zentrale Venenkatheter in der benachbarten V. jugularis interna einlagen, was zuvor als mögliche Limitation diskutiert worden war [20]), inkonklusive Ergebnisse fanden sich nicht. Zu dem gleichen Ergebnis war auch eine kleine prospektive Studie an 20 Patienten zur Anwendung der ECCD im Rahmen der IHA-Diagnostik gekommen [18].

Mittels TCCD war in unserer Studie bei einem Drittel der Patienten der Nachweis der Irreversibilität des klinischen Hirnfunktionsausfalls nicht möglich. Die intrakraniellen Gefäße waren bei 23 % (i.e. 26 von 114) gar nicht, bei 8 % (i.e. 9 von 114) nur teilweise darstellbar. In früheren Studien war dies bei 27–43 % der Fall[17, 22]. In unserer Studie war dies hauptsächlich auf komprimierte Gefäße infolge von massiv erhöhtem intrakraniellem Druck (ICP) oder auf verzerrte anatomische Verhältnisse aufgrund ausgedehnter zerebraler Läsionen oder Ödeme zurückzuführen, ein fehlendes temporales Schallfenster lag bei 10 Patienten vor. In einer retrospektiven Studie zur Anwendung der ECCD im Rahmen der IHA-Diagnostik hatte sich eine niedrigere Sensitivität als in unserer Studie gezeigt [16]. Frühere Studien diskutieren, dass die Beurteilung des ACI-Siphons mittels transorbitaler Dopplersonographie beim Fehlen eines ausreichenden temporalen Knochenfensters oder der Einsatz von Kontrastmittel in der vorgenannten Situation hilfreich sein könnten [11, 22]. Allerdings lässt sich mit der Dopplersonographie ohne direkte Sicht auf das Gefäß die Richtung des Blutflusses (und damit die Verteilung des ortho- und des retrograden Anteils des Blutflusses) nicht mit absoluter Sicherheit bestimmen [20]. Außerdem kann versehentlich die A. ophthalmica beschallt werden, und es kann zu einem Shunt des Blutflusses in das System der A. carotis externa kommen [8]. Der Einsatz der TCCD erfordert das Vorhandensein eines entsprechenden Schallkopfs und der speziellen Expertise bei der Anwendung dieser Methode insbesondere bei diesem Patientenkollektiv.

Bei 6 % (7 von 114) unserer Studienpatienten wurden mit der TCCD einzelne intrazerebrale Gefäße mit Diastole nachgewiesen, die häufig retrograd perfundiert waren. Dies könnte theoretisch auf extra-intra-kranielle Kollateralen zurückzuführen sein, d. h. über die A. carotis externa (ACE) und die A. meningea media. In allen 7 Fällen zeigte die im Rahmen der Studie durchgeführte ECCD ein zu einem Perfusionsstillstand passendes Flussprofil. Klinisch fand sich eine Hirnstammareflexie und das zusätzlich eingesetzte EEG zeigte über > 30 min eine Nulllinie an, was auch in früheren Studien so beschrieben wurde [4, 22]. Die Bedeutung des Nachweises eines solchen isolierten, nicht zum IHA passenden Flussprofils bei gleichzeitig passendem Flussprofil der übrigen zerebralen und auch der zuführenden extrakraniellen Gefäße ist aus unserer Sicht fraglich.

### Testgüte

Bei allen 94 Patienten mit Perfusionsstillstand im ECCD konnte der zum IHA passende Befund durch TCCD (*n* = 56) oder EEG (*n* = 38) bestätigt werden. Es gab keinen falsch-positiven Befund, also keinen Patienten mit Nachweis eines Perfusionsstillstands im ECCD und Restperfusion im TCCD bzw. Restaktivität im EEG.

Bei 20 Patienten zeigten im ECCD und auch TCCD eine Restperfusion, sodass kein Nachweis der Irreversibilität des klinischen Hirnfunktionsausfalls möglich war. Bei 15 dieser Patienten fand sich entsprechend auch Restaktivität im EEG, bei 5 zeigte sich hingegen ein zum IHA passendes Nulllinien-EEG. Insgesamt ergab dies eine Sensitivität von 0,949 und einen NPV von 0,75 bei einer Spezifität und einem PPV von 1. Dies ist vergleichbar mit Ergebnissen früherer Studien zur Anwendung der transkraniellen Dopplersonographie im Rahmen der IHA-Diagnostik [5].

Bei allen 5 Patienten mit Diskrepanz zwischen Duplexsonographie und EEG waren die klinischen Zeichen des IHA bereits > 72 h zuvor eingetreten, alle hatten eine sekundäre Hirnschädigung (Hypoxie). Deshalb ist anzunehmen, dass entweder primär eine komplette hypoxische Schädigung aller zerebralen Neurone bei noch erhaltener Restperfusion vorlag, wie in der Literatur vorbeschrieben, oder aber der ICP-Peak zum Zeitpunkt der Durchführung der Duplexsonographie bereits überschritten war und bereits wieder eine Reperfusion eingesetzt hatte [1]. Eine solche Reperfusion, gerade bei Patienten mit hypoxischer Hirnschädigung wird auch in der Literatur und der Richtlinie beschrieben [4, 17, 21, 22] und für diese Situation ein Methodenwechsel vorgeschlagen. Dies betrifft natürlich alle Perfusionsverfahren und nicht alleinig die Duplexsonographie in diesem Kontext, kann aber dann problematisch sein, wenn der Nachweis eines ZP (z. B. bei primär infratentorieller Hirnschädigung oder bei inkomplett beurteilbarer Hirnstammareflexie) unabdingbar ist.

Die Validität der CT-Angiographie im Kontext IHA-Diagnostik und Nachweis des ZP liegt in verschiedenen Publikationen zwischen 52 und 100 % [21], die der SPECT bei 75–100 % [2, 7, 15]. Der optimale Zeitraum zur Anwendung von ECCD/TCCD bei diesem Kollektiv ist unseres Erachtens 24–72 h nach dem Auftreten der klinischen Zeichen des IHA.

### Limitationen

Weitere mögliche Einschränkungen der Anwendung der ECCD im Kontext der IHA-Diagnostik bestehen darin, dass eine laufende ECLS-/vaECMO-Therapie oder Kalottendefekte den Nachweis der Zeichen eines zerebralen Perfusionsstillstandes erschweren oder diesen unmöglich machen. Hier hat die (zum Zeitpunkt der Studiendurchführung noch nicht gültige) aktuelle 5. Fortschreibung der Richtlinie der BÄK zum Nachweis des IHA [1] auf die fehlende ausreichende Validierung verwiesen und alle Verfahren, die auf dem Nachweis des ZP beruhen, als nicht geeignet bezeichnet.

Vorsicht ist außerdem geboten bei Patienten mit ausgeprägter supraaortaler Makroangiopathie und insbesondere Verschlüssen oder höchstgradigen Stenosen der distalen extrakraniellen ACI. In dieser Konstellation könnten Pendelfluss oder systolische Spitzen auch im Rahmen des Gefäßprozesses erklärt sein und es könnte ein Shunting über die ACE und die A. ophthalmica begünstigt werden. Um dies eindeutig abzugrenzen, wäre eine Untersuchung aller denkbaren, der ACE entspringenden Kollateralen vonnöten, was kaum realistisch ist. In solchen Fällen ist entweder die zusätzliche Durchführung der TCCD des intrakraniellen ACI-Abschnitts, oder ein alternatives Zusatzverfahren zu empfehlen.

### Alternativen

Alternative diagnostische Verfahren zum Nachweis des zerebralen Perfusionsstillstands (CTA, DSA, SPECT) sind deutlich komplexer, im Falle der CTA und DSA ist eine zusätzliche Kontrastmittelgabe nötig, und alle vorgenannten Verfahren machen den Transport kritisch kranker und damit potenziell kardiopulmonal instabiler Patienten nötig. Weiterhin ist das richtlinienkonforme Protokoll für diese Prozeduren komplex und voller Fallstricke und auch bei manchen Patientengruppen gar nicht zugelassen (z. B. CTA bei Patienten < 18 Jahre). Die DSA ist in Deutschland nur für ein sehr umschriebenes Patientenkollektiv zum Nachweis eines ZP zugelassen und zwar dann, wenn sie primär zur Klärung der Art der Hirnschädigung bzw. zur Therapieentscheidung durchgeführt wurde. Zeigt sich in diesem Zusammenhang ein ZP, so ist die Irreversibilität des Hirnfunktionsausfalls damit bestätigt [1, 14].

Geprüft werden sollte der Einsatz von Ultraschallkontrastmittel, welcher richtlinienkonform möglich ist und die Darstellbarkeit der intrakraniellen Gefäße bei IHA-Kandidaten verbessern kann [13, 22]. Trotz des Einsatzes von Kontrastmittel waren in den genannten Studien aber auch dann nicht in allen Fällen alle geforderten Gefäße darstellbar und bei fehlendem temporalem Knochenfenster ist diese Alternative ebenfalls wenig hilfreich. Die Richtlinie gibt für die CCD vor, dass das „Fehlen der Strömungssignale bei transkranieller Beschallung der Hirnbasisarterien als sicheres Zeichen eines zerebralen Kreislaufstillstandes gewertet werden kann, wenn derselbe Untersucher bei gleicher Geräteeinstellung bei einer früheren Untersuchung eindeutig ableitbare intrakranielle Strömungssignale dokumentiert hat oder wenn an den extrakraniellen hirnversorgenden Arterien die Zeichen des zerebralen Kreislaufstillstandes nachweisbar sind.“ Das lässt offen, ob dann generell eine reine Untersuchung der extrakraniellen hirnversorgenden Gefäße ausreichend ist oder aber auch nur einzelne Stromgebiete, für die intrakraniell kein Strömungssignal abgeleitet werden kann, durch die Ableitung des weiter proximal gelegenen extrakraniellen Gefäßabschnitts ersetzt werden können.

## Fazit

Die TCCD war in unserer Studie in einem Drittel der Fälle für sich alleine genommen nicht aussagekräftig, um den ZP und somit die Irreversibilität des klinischen Hirnfunktionsausfalls nachzuweisen. Im Gegensatz dazu war die > 24 h nach Auftreten der klinischen Symptome des IHA eingesetzte ECCD bei allen Patienten einfach und bettseitig durchführbar. Sie kann auch mit Standardultraschallgeräten mit Linearschallkopf und ohne spezielle TCCD-Kenntnisse und -Sonden durchgeführt werden. Hauptlimitation ist der nicht zu empfehlende Einsatz bei Patienten mit ausgeprägt stenosierender Makroangiopathie oder Verschluss insbesondere der ACI.

In einer prospektiven, multizentrischen Studie sollte geprüft werden, ob bei allen anderen Patienten das Fehlen falsch-positiver Ergebnisse in dieser Studie bei Anwendung > 24 h nach dem Auftreten der ersten klinischen Symptome des IHA (Spezifität von 1) möglicherweise den alleinigen Einsatz der ECCD als Alternative zu den aktuell zugelassenen Verfahren erlaubt. Dies ist im Rahmen des IGNITE!-Netzwerkes der DGNI bereits geplant. Einstweilen können die Ergebnisse dieser Studie als Bestätigung des in der Richtlinie (5. Fortschreibung) genannten Vorgehens zum ausnahmsweisen Einsatz der ECCD im Falle fehlender Flusssignale bei Beschallung der Hirnbasisarterien gewertet werden.

## Abkürzungen

ACE: A. carotis externa
ACI: Arteria carotis interna
ACM: Arteria cerebri media
BA: Arteria basilaris
BÄK: Bundesärztekammer
CCD: Farbkodierte Duplexsonographie
CCT: Kraniale Computertomographie
COPD: Chronisch obstruktive Lungenerkrankung
CPR: Kardiopulmonale Reanimation
CTA: CT-Angiographie
DGKN: Deutsche Gesellschaft für Klinische Neurophysiologie
DGNI: Deutsche Gesellschaft für Neurologische Intensivmedizin
DSA: Digitale Subtraktionsangiographie
ECCD: Extrakranielle farbkodierte Duplexsonographie
ECLS: Extracorporeal life support
EEG: Elektroenzephalogramm
ICB: Intrazerebrale Blutung
ICP: Intrakranieller Druck
IGNITE!: Initiative of German NeuroIntensive Trial Engagement
IHA: Irreversibler Hirnfunktionsausfall
IQR: Interquartilsabstand
MRT: Magnetresonanztomographie
NPV: Negativer prädiktiver Wert
PPV: Positiver prädiktiver Wert
SAB: Subarachnoidale Blutung
SHT: Schädel-Hirn-Trauma
SPECT: Single-Photon-Emissionscomputertomographie
TCCD: Transkranielle farbkodierte Duplexsonographie
TCD: Transkranielle Dopplersonographie
VA: Arteria vertebralis
VaECMO: Venoarterielle extrakorporale Membranoxygenierung
ZP: Zerebraler Perfusionsstillstand
